# Nurses and the disabled child’s perspective in the anaesthesia procedure preparation process using a picture schedule

**DOI:** 10.1080/17482631.2024.2356927

**Published:** 2024-05-27

**Authors:** Johanna Kaitsalmi, Tanja Vehkakoski, Liisa Karlsson, Sanna Salanterä

**Affiliations:** a Department of Nursing Science, University of Turku, Turku, Finland; b Department of Education, University of Jyväskylä, Jyväskylä Yliopisto, Finland; c Faculty of Educational Sciences, Department of Education, University of Helsinki, Helsingin Yliopisto, Finland

**Keywords:** Agency, alternative and augmentative communication, applied conversation analysis, disabled children, nonverbal communication, nurse–patient relationship, participation, preparing children

## Abstract

**Purpose:**

This study’s purpose was to investigate how nurses, using a picture schedule, enable or hinder the realization of disabled children’s agency in the preparation for an MRI procedure carried out under general anaesthesia.

**Methods:**

A qualitative observation study was used to explore the interaction of nurses and children. The data consisted of video recordings of 25 preparation situations of 3 (3–8 years old) children (with challenges in communication and/or cognitive skills) with 4 nurses. Verbal and nonverbal communication was analysed with interventionist applied conversation analysis.

**Results:**

What was most crucial was how the picture schedule was used during the interaction. Reciprocal information sharing, responding to the child’s initiatives by negotiating and allowing the child to take physical action with the picture schedule enabled the realization of the child’s agency.

**Conclusions:**

The preparation process should aim to help the child prepare in his/her own way. The preparation tools should encourage reciprocal interaction in informing and in responding to the children’s initiatives. The preparation practices should include enough time for the child’s initiatives and physical participation. The results can be used in assessing preparation tools and how they are used from the perspective of the child’s agency.

## 1. Introduction

According to children, coming to hospital for a medical procedure is a distressing experience (Wennström et al., 2008). Even one “small” anaesthesia procedure has been shown to result in separation anxiety, aggression towards authority and other problems in children even one year later (Kain et al., 1996). Reducing children’s anxiety through preparing them mentally for the procedures has been studied extensively (see reviews, e.g., Capurso & Ragni, 2016; Dai & Livesley, 2018; Copanitsanou & Valkeapää, 2013) but most studies have left out the perspective of the children themselves. For example, of the 44 studies in Capurso and Ragni’s (2016) review, only three included the children’s own perceptions. Especially young children and children with communication problems have usually been ignored in the studies, or only their parents have been interviewed (see Capurso & Ragni, 2016). The perspective in previous studies has mostly focused on the actions of the professional. The commonly used expression “preparing children” refers to an image of a child where the child is seen as a passive object of care, instead of an active subject who could be “helped to prepare” for procedures.

Alleviating children’s anxiety during their hospital visit is an important nursing objective, but there are also other important aims from the children’s perspective. As Lindberg and von Post (2005, 2006) and Wennström et al. (2008) have shown, by helping children prepare themselves for a procedure, children may arrive happily for the procedure and feel proud of themselves during the procedure and afterwards. These consequences could also be called children’s empowerment (Mitcheson & Cowley 2003). In these studies, empowerment was achieved through a process called Perioperative Dialogue, which is based on listening to the children and sharing information in a dialogical interaction (Lindberg & von Post, 2005, 2006). Therefore, how to listen to children, also when they express themselves nonverbally, is an important question in helping children prepare for a medical procedure. In addition to empowering them, actively listening to children also enables the actualization of their right to be heard and have their perspectives considered (UN, 1989).

In this study, we are interested in the right of the child (especially the disabled child) to be heard (UN, 1989) in the anaesthesia procedure preparation process. Previous studies on disabled children’s hospital experiences show that everything intertwines with communication, which has been found to be challenging for nurses when interacting with disabled children (Hemsley et al., 2014, Sharkey et al., 2016; Oulton et al., 2015, 2018; Shilling et al., 2012; Simmons et al., 2019; Thunberg et al., 2015; Thunberg et al., 2016). Encountering a disabled child as an agentive patient is often more challenging for healthcare professionals than it is with typically developed children (Solomon et al., 2016). Our purpose in this study was to find nursing practices which enable the realization of the agency of children with developmental disabilities in the preparation process. The context of our study is a children’s neurological ward where nurses use a picture schedule in the preparation for a Magnetic Resonance Imaging (MRI) procedure carried out under general anaesthesia. The research question is: How do nurses enable or hinder the realization of the agency of children when preparing for an anaesthesia procedure with the picture schedule?

### 1.1. Children’s agency in healthcare

Every child has, according to the world’s most largely ratified human rights convention, the right to be heard in all matters affecting the child (UN, 1989), regardless of their age or skills (UN, 2009). However, only listening to the child is not enough; the views of the child have to be seriously considered (UN, 2009), that is, his/her agency should be realized. Agency is understood in this study as every human being’s need to have an influence on other human beings through communication, and the realization of agency as taking that need into account and responding to it appropriately. We differentiate between the concepts of “actorness” and “agency” as Mayall (2002) does: an actor is just someone who acts, but an agent is someone whose interaction makes a difference (Olli et al., 2012; Karlsson, 2020). The realization of the child’s agency does not, however, mean that the child always automatically gets what she/he wishes, but that his/her expressions of wishes are validated and taken into account. The child’s right to be heard and taken seriously is considered an intrinsic value, because seeing agency as an instrumental value may give adults too many opportunities to speculate about who will benefit from it and who will not (Olli et al., 2012).

Earlier research has, however, shown many good consequences for disabled children of being heard. The realization of children’s agency in the healthcare context may strengthen their experience of being normal (Bekken, 2014), their self-confidence (Lightfoot & Sloper, 2003; Mandich et al., 2003) and their sense of belonging to a community (Mandich et al., 2003). In addition, Lindberg and von Post (2005, 2006) as well as Wennström et al. (2008, 2011) have demonstrated the short-term consequences of using a process based on listening to children to help them prepare. In these cases, not only were the children’s stress and pain lower (Wennström et al., 2011), but also the children’s experiences of themselves and the whole hospital process were better than expected (Lindberg & von Post, 2005, 2006; Wennström et al., 2008). In Lindberg and von Post’s (2005, 2006) the child participants were chosen specifically because they had had previous bad experiences at hospital. When these children came to the procedure after the preparation process, they were confident: they knew they could manage their own part, and they were ready to trust themselves in the hands of professionals—they even enjoyed coming back to the hospital.

The possibilities for the realization of children’s agency in hospital are related to the professionals’ image of a child (Karlsson, 2020). Traditionally in healthcare, the image of a child has typically been one that emphasizes children’s vulnerability and need for protection (Olli et al., 2014). The problem with this kind of thinking is that it ignores the child’s right to participate in decisions concerning his/her life (Bekken, 2014), and children may be protected even from themselves because of their insufficient cognitive skills. Instead, if children are seen as active social agents who are able to influence their own life’s decisions (James & James, 2004; Mayall, 2002), then listening to children would be a starting point for professional practices and would always be present in some form (Karlsson, 2020).

The realization of children’s agency in hospital is also related to the professionals’ ability to communicate with children. Previous literature review (Olli et al. 2012) has demonstrated that communication practices based on dialogical interaction enables the realization of disabled children’s agency. Dialogical communication theories emphasize the idea of equality of interaction partners, for example describing it as “I-Thou” relationship differentiating it from “I-It” relationship where the other one is seen as an object (Buber & Smith, 2004). Essential in dialogicality is also regarding both communication partners as both ignorant and knowledgeable at the same time and, therefore, trying to learn together from each other (Freire & Ramos, 2005). In dialogical communication neither of communication partners can know the outcome of the communication beforehand, since it is created together (Olli et al. 2021).

### 1.2. The use of alternative and augmentative communication for enabling children’s agency in hospital

Since children of all ages and with every kind of communication skills have the right to be heard, communication other than just verbal is required from adults (UN, 2009). Alternative and augmentative communication (AAC) includes high-tech aids (such as applications for digital devices, voice output aids or eye-tracking devices) as well as low-tech or even no-tech aids (such as pictures, objects or manual keyword signs). There are only few studies on using AAC with children in hospital. However, according to Hemsley and Balandin’s (2014) review, children’s basic communication needs are similar to adults’, and therefore, the research results of adult’s AAC communication can also be applied to children. One observation concerning disabled children is that they want to communicate directly with hospital staff (Hemsley et al., 2013), but professionals have been found to speak mostly with the parents (Hemsley et al., 2013, Hemsley et al., 2014; Oulton et al., 2015; Sharkey et al., 2016; Thunberg et al., 2016).

Earlier research has indicated a clear discrepancy between nurses and patients with complex communication needs in what they think is satisfying communication. According to Karlsen et al. (2018) review, nurses tend to think that they understand the patients better than the patients feel that they were understood. Views on what is important to patients also differ clearly between nurses and patients (Karlsen et al., 2018). In order to be able to express their own views, patients have described a need for easy-to-use communication aids, and they have been more satisfied with their care if AAC has been used (Karlsen et al., 2018). That might be linked to the finding that professionals tend to communicate more often and longer with patients when using AAC (Happ et al., 2004; Nilsen et al., 2013). The use of AAC has also proven to save time and ease frustration in communication (Hemsley & Balandin, 2014). Yet healthcare professionals are not often active in using AAC even when they see it is important and useful (Handberg & Voss, 2018).

Pictures as a communication method in hospital have not been studied from children’s perspectives. Disabled children’s parents have, though, wished for more use of pictures with children in hospitals (Sharkey et al., 2016; Thunberg et al., 2016). Healthcare professionals in a pilot study evaluated that pictures enabled communication with children and helped some children’s participation (Thunberg et al., 2015). According to professionals, pictures added playfulness to the hospital visit, as children were interested in the pictures and perceived them as fun and exciting (Thunberg et al., 2015). Professionals also found that using pictures as tools for structuring time or activities helps children in concentrating and in anticipating what is going to happen and, therefore, gives children a feeling of control over a situation (Thunberg et al., 2015). In addition, using a picture schedule has significantly lowered the distress of children when being helped to prepare for procedures (Chebuhar et al., 2013; Vantaa Benjaminsson et al., 2015).

The flip side of structured picture schedules is that they might make professionals less flexible (Thunberg et al., 2015) and weaken the opportunities for the patient to participate (Mayor and Bietti, 2017). According to Mitcheson and Cowley (2003), a structured method may even be harmful, since it gives power to the nurse and hinders listening to the patient.

In summary, using AAC for communication and/or structuring may offer both opportunities and threats for supporting the realization of disabled children’s agency. In our study, we explored both these aspects of using AAC by analysing video-recordings of the interactions of nurses and children through applied conversation analysis.

## 2. Materials and methods

### 2.1. Design, setting, participants and data collection

The study setting of this qualitative observation study was a children’s neurological ward in a public special healthcare hospital in Finland. The data consisted of video-recordings of fourteen preparation situations where four nurses used a picture schedule (Picture 1) with three children during their MRI visits. Typical case sampling (Patton, 2002) was used for obtaining widely applicable knowledge when selecting children with a common diagnosis for patients in neurological wards: F83, “mixed specific developmental disorders” (ICD-10, 2016). The children had challenges with communication (such as inarticulate speech or problems with forming sentences) and/or cognitive skills (such as comprehension or attention deficits), but they were all able to understand speech and to speak to some extent. Yet focusing on many things at the same time (e.g., the picture schedule and the other’s multimodal communication) might be difficult, and the attention deficit might cause problems in digesting long explanations. The frightening or otherwise stressful situation might weaken the child’s comprehension or expression even more. Maximum variation sampling (Patton 2002) was used for obtaining rich data when selecting children of different ages (three-, five- and eight-year-old boys) and nurses with short (2.5 years) to long (23 years) experiences in paediatric nursing.

**Figure:**
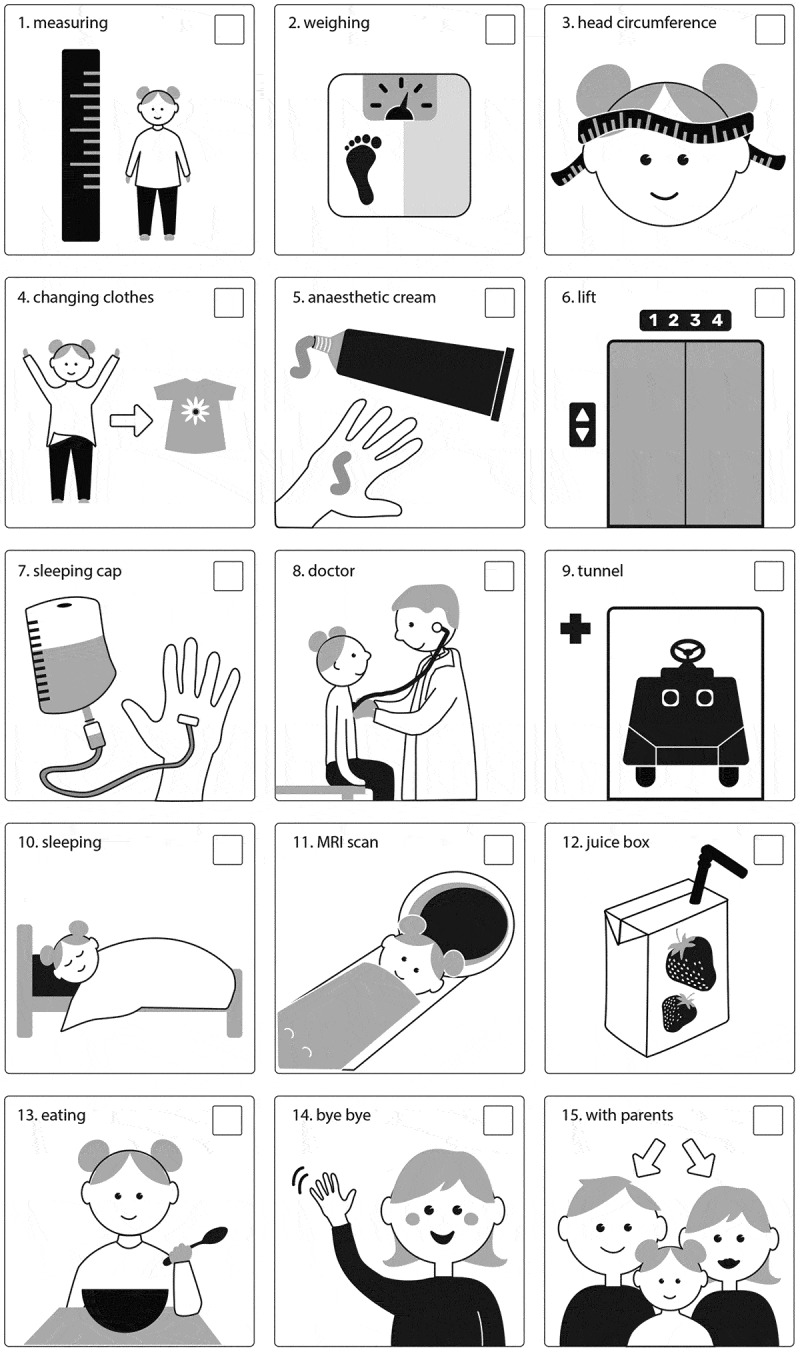
Picture 1. the picture schedule in the beginning of the day.

The children visited the ward one time for 6–9 hours with their parents for an MRI scan. The nurses prepared the children with a picture schedule for the activities of the day when they arrived, and also between every action. The schedule was used during 7–9 sequences (from 26 seconds to 18.25 minutes) per child, constituting 25 preparation situations and 58.15 minutes of data in total. The first author collected data with a video recorder.

The picture schedule was made by the nurses working in the ward where the study was implemented. They had been using the schedule several months before the study without any specific training for using it, since using AAC was familiar to them. The schedule was a laminated A4 paper with fifteen pictures about the activities of the hospital visit (Picture 1). The idea of the schedule was to visualize the activities for the children and give them a chance to participate by letting them cover a picture after every activity with a “done” tag that had velcro tape on it (Picture 2). The titles of pictures were written in line with spoken language nurses used with children, e.g., infusion drip (picture 7) was called “sleeping cap”.

**Figure:**
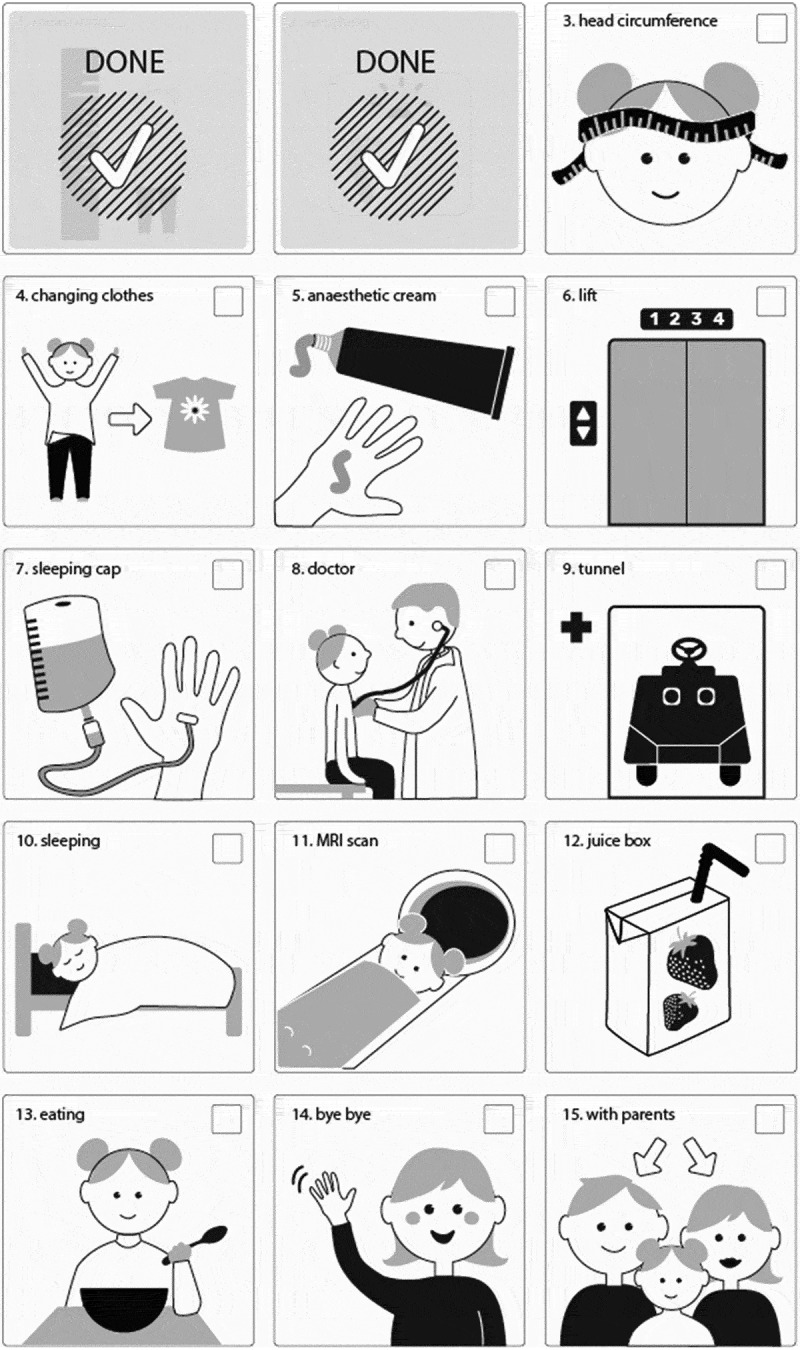
Picture 2. The picture schedule after the first two activities have been covered with a “done” tag.

### 2.2. Analysis

The data were analysed with an applied version of conversation analysis (CA). The analysis was based on traditional CA’s main idea, which is that the responses of the participants reveal their interpretations of the preceding turn of the other participant (Heritage, 2001). We used an interventionist applied version of CA because it is suitable for finding solutions for problems in interaction, and therefore, the analysis can be viewed from the professionals’ or the clients’ perspective (Antaki, 2011). We also wanted to use accessible language that can be understood by practitioners working with disabled children and potentially even by a wider audience. Therefore, we did not use the terminology and the detailed transcription conventions typical of traditional CA which is “readable” only by academics trained in that specific method (O’Reilly et al., 2020).

Conversation analysis has much to offer for nursing interaction research, since it unpacks the details of nursing interaction (Dowling, 2007; Jones, 2003; Mayor and Bietti, 2017) and also pays attention to multimodality, which has very seldom been analysed in nursing research (Mayor and Bietti, 2017). In our study, analysing the multimodal interaction was essential, since in addition to speech, the children also interacted extensively through nonverbal modalities such as facial expressions, touch and bodily movements. In addition, the nurses used the schedule as a visual and material resource.

The preliminary analysis was based on a rough transcription of all the data concerning the use of the picture schedule during the children’s MRI visit. The focus of the analysis was on the interaction practices that either enabled or hindered the realization of the child’s agency, i.e, whether the child’s communication made a difference in the nurse’s communication or not. We found six interaction practices which were related to three preparing activities. After this, we selected the richest examples of all types of practices and carried out a sequential analysis through paying attention to the turn-taking between the nurses and children as well as to the responses to the children’s verbal and nonverbal initiations. The analysis was made on the basis of the detailed transcriptions of the video-recordings as well as repeated views of the video recordings. The transcription conventions used are included in Appendix 1. The activities of the child or adults, which happen simultaneously with speech, are placed inside double brackets. The start of the activity is indicated under the word spoken at the same time.

### 2.3. Ethics

The basis of the study was respecting children’s right to be heard and protected. Therefore, we did not adopt an “ethnomethodological indifference” traditional for the original form of CA (see Dowling, 2007), but took the client’s point of view, as is suitable in interventionist applied CA (Antaki, 2011). We evaluated the nurses’ actions from the perspective of disabled children’s agency.

We asked for written informed consent from the nurses and the guardians of the children, but we also asked the children to give their assent with the help of personalized picture information sheets and assent or dissent pictures. Meeting the children before data collection enabled children to be in an active agent position which contributed to respecting the children’s perspectives in the analysis and preparation of the manuscript. The children were also provided with “stop videotaping” picture for discontinuing the participation but none of them used it (see for more detail Olli, 2019).

The children’s and nurses’ names are pseudonyms, and the picture schedule (picture 1) has been drawn for this article (according to the original) to protect the anonymity of the ward where the original is used. To promote the realization of children’s rights, we will later deliver pictured study reports to the child participants, in addition to the reports to the adult participants. The study was approved by the ethical committee of University of Turku, and permission from the hospital authorities was granted. The ethical principles of the Declaration of Helsinki were respected throughout the research process.

## 3. Results

The realization of the child’s agency in a preparation process for an anaesthesia procedure was related to the following three preparing activities: 1) informing the child, 2) responding to the child’s initiatives and 3) controlling the child’s physical actions with the picture schedule. The characteristics of the picture schedule as well as the way in which the nurses used the schedule had a pivotal role in hindering or enabling the realization of the children’s agency.

### 3.1. Informing the child in a reciprocal or nonreciprocal manner

Providing information is a central part of helping a child prepare for a procedure. An important question when providing information is whether the child has an opportunity to influence the content of the information and how it is shared. In the following extract (Table 1), the child’s initiatives direct the interaction between the nurse and the child. “Tom” is 5 years old and has “mixed specific developmental disorder” as diagnosis. He is very talkative, but his pronunciation was partly unclear. He understands speech as others in his age, but he has problems with keeping attention focused. The extract is from the beginning of the hospital visit, when the nurse (“Sofia”) and Tom are looking at the picture schedule, and the nurse has already presented the first eleven pictures. Tom interrupts the nurse’s presentation by asking about the tunnel in the ninth picture, which the nurse mentioned seven seconds previously. This starts a 1.14-minute sequence of talk.

**TABLE 1.** Data extract: reciprocal information sharing.

1	Tom	what is that tunnel? ((points at the picture of a forklift in a tunnel))
2	Nurse	it’s a sort of long tunnel there underground and there those kinds of forklifts ((points at the forklift/tunnel picture))
3		are running (.) you’ll see then (.) it’s a fine place (.) we’ll go there with this bed ((points at the bed))
4	Tom	[how?
5	Nurse	have you ever] been there?
6	Tom	how?
7	Nurse	we’ll go with the bed in a such way that we will push this bed with nurse Maija
8		and mum is with us
9	Tom	oh are we going underground? ((walks a few steps away and rubs his eye with one hand))
10	Nurse	well yes we’ll go you know along a sort of corridor (.) yes (1.3) we have long
11		corridors there underground (1.1) have you ever been in one? ((leans to the child))
12	Tom	no (.) I (.) want to see (.) where they are ((looks at the toy figure in his hands))
13	Nurse	they are corridors (.) just like the ones we have over here (.) just here (.) this ((walks to the door, guides Tom by the shoulder)) ((Tom starts walking with the nurse)) ((points to the corridor))
14		kind of corridor (.) below ground is that kind of long corridor
15	Tom	is there sand all around?
16	Nurse	well there can’t be sand really (.) since they are hospital corridors (.) ((Tom walks along the corridor twisting and turning))
17		but there you can drive with that kind of big forklift (.) mmm ((goes back to the room with Tom))
18	Tom	@with a forklift@
19	Nurse	yee:ah (.) hopefully we’ll see one of those (.) look then (.) Tom (.) what then (.) ((shows the picture schedule to Tom))
20		then when you have slept well (.) so then you’ll get a juice box

This extract begins with Tom asking what the tunnel is and pointing to the picture of the forklift in a tunnel. This starts a 1.14-minute conversation. The nurse’s a answer represents a preferred response to the child’s question, since she orients to the child’s interest by describing the tunnel, making it inviting to Tom, and by telling him more about the transition (lines 2–3). After the nurse’s explanation, Tom asks his second question: *how*? (line 4). At the same time, the nurse asks if Tom has ever been in the hospital tunnels. Since the nurse and Tom start talking simultaneously, neither of them answers the other’s topic initiations, but Tom repeats his third question emphatically: *how*? (line 6). Now the nurse aligns with Tom again by explaining how and with whom they will go into the tunnel.

Tom continues by giving a candidate understanding about the nurse’s explanation in the form of the following fourth question: *oh are we going below ground*? The nurse confirms Tom’s turn and describes how they will go down the corridor. Through the 1,3 second pause in the middle of her turn she also enables Tom’s involvement, but when Tom does not take the turn, she mentions the hospital’s long corridors underground, but does not give any new information. Then the nurse repeats her earlier question about whether Tom has been in the tunnel before. Tom’s answer is negative, but he expresses a wish to see where the tunnels are. The nurse orients to Tom’s wish when demonstrating the meaning of it more concretely: she walks to the corridor and invites Tom along to see how the underground corridors are similar to the one they are looking at.

Tom continues leading the conversation with his fifth question, which reveals something about his image of the tunnel: *is there sand all around?* (line 15). The nurse explains that this is not possible in a hospital and takes the conversation back to an earlier topic, the forklifts. Tom repeats *with the forklift* (line 18) in a pensive tone, which the nurse takes as a signal of interest (line 19). The nurse also takes Tom’s utterance as a signal for being ready to move on to the next topic with the picture schedule.

In summary, in this extract, the child takes a very active role in finding out what the nurse is talking about. He asks four different questions and repeats the one that does not get an answer right away. Although the picture schedule works as a starting point for the discussion, it does not include pictures for more specific questions or explanations. As a response to the child’s questions, the nurse explains the subject more specifically and visualizes a similar place. The schedule enabled the child to initiate the conversation, although the nurse had already passed the picture in which he was interested.

In contrast to extract 1, the preparing process sometimes entails only giving information, not developing into a reciprocal conversation. In extract (Table 2) below, the nurse (“Sofia”) has just asked the child (“Niko”) to come and look at the picture schedule. Niko is 8 years old and has “mixed specific developmental disorders” as diagnosis. He is able to talk about everyday items as well as others in his age, while complex concepts are more difficult to him, and he is very quiet with the nurse during all day. To other people he talks easily. Sofia itemizes the activities of the day without allowing the child to comment or ask any questions. Niko, his father and the nurse sit on a sofa to look at the picture schedule. Niko sits without moving through the whole 46-second episode with a serious face and follows with his gaze the pictures that the nurse is pointing to.

**Table 2.** Data extract: Nonreciprocal information giving.

1	Nurse	look (.) we’ll measure how tall you are (.) much you weigh (.) we’ll ((points at the weighing picture)) ((glances at Niko who is looking at the schedule))
2		measure the head circumference (.)
3		then we change our clothes for a moment(.) ((points at the picture, looks at Niko))
4		then we put anaesthetic cream a little here (.) ((touches the back of Niko’s hand))
5		then we go to the elevator (.) then we put the sleeping cap (.) ((touches the back of Niko’s hand))
6		then our doctor listens a little here (.) ((touches Niko’s chest))
7		then we’ll go to this kind of tunnel (.) where we can see these kinds of ((points at the tunnel picture)) ((glances at Niko))
8		forklifts
9		then you can sleep (.) here is the examination and also there you only sleep (.) ((points at the sleeping picture))
10		then when you have woken (.) you’ll get a juice box and food (.) ((points at the juice box picture)) ((points at the food picture))
11		then we’ll say bye (1.57) that kind of day we’ll have (.) ((stays looking at Niko)) ((Niko is barely noticeably nodding his head))
12		isn’t it quite a nice day?
13		(1.04)
14	Niko	((nods his head in a barely noticeable way))
15	Nurse	yeah (.) and nothing else will happen (.) here is what will happen to us today ((looks at the schedule, draws a circle in the air above the schedule))

The first part of this extract is the nurse’s long turn where she tells Niko about the activities of the MRI day. The day consists of fifteen activities, which the nurse lists one after another. The listing repeats the same form: the nurse uses the particle “then” as a transition word between the previous and the following picture, and either points to the picture in the table (lines 3, 7, 8 and 9) or touches the child in order to demonstrate the activity (lines 4, 5, 6).

The nurse presents the pictures with pauses that are shorter than one second. These kinds of pauses might possibly be interpreted as opportunities for the conversation partner to take their turn in reciprocal conversations between equal conversation partners. However, the nurse glances at Niko only when speaking, not when pausing, which is when she looks at the schedule. This refers to the pauses serving as transitions from one picture to another rather than providing a turn for Niko who doesn’t take the turn during the pauses. After mentioning the last activity *then we’ll say bye* (line 10), she pauses for 1.57 seconds and looks at Niko, thus giving him an opportunity to respond. When Niko does not quickly take his turn during this short pause but looks continuously at the schedule, she completes her narration by means of a closing summary *that kind of day we’ll have* and asks a closed-ended question to search for an agreement from Niko: *isn’t it quite a nice day* (line 10). Niko answers as expected by nodding slightly. After getting the answer, the nurse states that she has told him everything that will happen. Then, she closes the preparation situation and leads Niko to the first activity.

In summary, this extract describes a nurse-led situation where the nurse is primarily oriented to giving information and structuring the day for the child. The child looks only at the schedule all the time, and he is not encouraged to participate as an active agent in this possibly strange situation containing much information delivered in a short time. We do not know if any other communication resources would have encouraged the child, since the nurse does not try to use any other strategies, such as asking the child to freely share his thoughts and feelings or encouraging him to ask questions or point to the pictures. The picture schedule is used only as a visualization of her narration, not as a tool for the child to participate in the conversation or to manage his emotions.

### 3.2. Responding to the child’s initiatives by negotiating or by sticking to the structure

The picture schedule is typically used for making visible the quite rigid structure of actions during the MRI visit. Before each transition, the nurse shows the next picture in the schedule to the child and mentions what they will do next. After the activity in question, the picture of that activity will be covered with a “done” tag. However, sometimes the schedule might be used for discussion. In extract (Table 3) below, at the beginning of the hospital day, the nurse uses the schedule in a flexible way to respond to the child’s facial expression. Before the situation, the nurse (“Heidi”) has already presented most of the schedule to the child. “Jani” is 3 years and has “mixed specific developmental disorders” as diagnosis. He speaks very little, mostly with 1–2 words sentences and his pronunciation is very unclear. Pictures are important to him, and he is used to using them in his everyday life. Jani’s mother holds him by the hand. Jani looks at the schedule the entire time (if not stated otherwise).

**TABLE 3.** Data extract: Responding to the child’s initiatives by negotiating.

1	Nurse	and then when you’ll come back here so then (.) then you’ll get a juice box ((points at the juice box picture)) ((Jani grimaces so that his upper teeth show)
2		and after it food (.) and then the day is almost over and we can say bye
		**((17 seconds of text excluded. The nurse continues presenting the structure.))**
3	Mother	Jani doesn’t like that juice box so= ((Jani looks to his side)) ((Jani looks to the schedule))
4	Nurse	=okay (.) so then some other juice
5	Mother	it probably [scares a little when he thinks he has to drink= ((Jani looks down))
6	Nurse	yeah]
7	Nurse	=you don’t have to take (.) we can [hide it from there (.) ((attaches a done tag on top of the juice box picture))
8	Mother	water (.) water (- -)]
9	Nurse	let’s hide the juice box picture (.) you’ll get something else then (.) so now ((Jani looks to the schedule)) ((Jani looks to his side))
10	Nurse	look it is hidden ((passes the schedule nearer Jani))
11	Mother	finished (.) now it’s finished (.) no need to take a [juice box ((Jani looks to the schedule)) ((mother points at the covered picture)) ((Jani looks at the schedule and has a hint of a smile on his face))
12	Nurse	don’t need to take]

In this extract, the nurse is telling Jani about getting a juice box after the MRI. As an immediate response, Jani displays his emotions with a facial expression, grimace. However, the nurse does not either perceive this or ignores it, and continues her explanation. After 17 seconds, Jani’s mother returns to the topic and verbalizes Jani’s grimace. This gets the nurse to suggest replacing drinking a juice box to drinking another kind of juice. The mother continues to speak on behalf of Jani by explaining that the thought of drinking might scare Jani. As a response, the nurse smooths over the situation through an acknowledgement token (line 6) and a verbal promise (lines 7, 9 and 12), and by the action of covering the juice box picture with a “done” tag. After the nurse and mother’s shared reassurance, Jani looks at the schedule and has a hint of a smile on his face.

In summary, this extract describes a situation where the child’s response (interpreted by his mother) is considered a relevant initiative for a discussion. Although the picture schedule did not enable removing the unwanted picture, the nurse’s creativity allows using the schedule to respond to the child anyway.

Contrary to extract 3, in the following extract (Table 4), the child’s initiative is taken as an error that should be corrected. The child (“Jani”) does not follow the idea to cover the pictures of the past activities with the tags, but covers activities that have not yet been done. The extract describes a situation where the nurse (“Heidi”) and the child (and his mother) look at the picture schedule after the nurse has described the idea of “done” tags to Jani.

**TABLE 4.** Data extract: Responding to the child’s initiatives by sticking to the structure.

1	Nurse	what have we done already?
2	Mother	what was finished?
3	Nurse	do you want to put this by yourself? ((passes done tag to Jani))
4		((Jani takes the tag, looks at the back side of it))
5	Nurse	there’s that kind of velcro tape (.) I can help you a little ((points at the schedule, then points at the tag))
6	Jani	((attaches the tag on top of the MRI picture))
7	Mother	that we haven’t do[ne yet
8	Nurse	we] haven’t done that yet (.) have we measured you yet? ((point at the measuring picture))
9	Jani	have measured
10	Mother	[have measured (.) put it there on the top of
11	Nurse	have measured (.) will we put it there on top of] (.) let’s put it the- (.) ((starts removing the tag)) ((Jani takes the tag from the nurse’s hand and attaches it on top of the measuring picture))
12		well done you
13	Mother	finished [(.) finished
14	Nurse	good]
15	Mother	that we won’t do anymore (.) [it is finished
16	Nurse	then (.)] have we weighed yet? ((takes another tag from the box and points at the second picture)) ((passes the tag to Jani))
17		((Jani takes the tag, looks at the back side of it, attaches it on top of the sleeping picture))
18	Mother	put it there on top of the scales
19	Nurse	sleeping (.) you have probably been sleeping at night (.) but that is like ((removes the tag, even if Jani holds on to it, and moves it on top of the weighing picture))
20		an afternoon nap picture
21		so will we put it there (.) do you wanna put it yourself (.) there you go ((Jani attaches the tag on top of the weighing picture))

In extract 4, the nurse starts with asking Jani what they have already done (line 1), followed by the mother reformulating the question (line 2). However, after receiving detailed instructions for the activity, Jani does not attach the tag to the first picture, but to the MRI picture, which is the eleventh picture in the table (line 6). As a response, both adults correct Jani explicitly in lines 7 and 8: an MRI has not been done yet, and Jani has thus covered the wrong picture. In addition, the nurse continues by moving Jani’s attention to the right picture by pointing at the first picture and posing a close-ended question: *have we measured you yet?* (line 8). Jani’s positive answer claims understanding of the question and of what has happened (line 9). He also attaches the tag to the picture expected by the nurse, and both adults confirm this as the right way with agreement tokens, praise and explaining phrases (lines 11–15).

The nurse’s next close-ended question (line 16) is related to the second picture (measuring), and it is accompanied with pointing to the picture. However, Jani covers the sleeping picture, which is the tenth picture. His mother corrects Jani (line 18) by prompting him to cover the scales picture, and the nurse interprets Jani’s action as a way of talking about sleeping during the night before. Jani does not change the place of the tag and tries to hold it, but the nurse takes it from his hand and gives Jani the opportunity to attach it in the place she sees as correct.

In summary, this extract is an example of a situation where the child has a different idea of the use of the picture schedule than the adults have. The adults do not orient to the possibility to use the schedule as anything but creating a visual structure (even though in extract 3 the same nurse covered the picture that the same child would not need to do). The nurse’s interpretation of the child’s thinking about sleeping the night before reflects the same idea of only covering the activities that have been done. The same interpretation did not, however, apply to covering the MRI picture, but the adults did not ask the child about his ideas. Closed-ended questions don’t help the child express his own ideas. The child’s way of using the schedule is constructed as an error, not as a relevant initiative for discussion. Therefore, the child doesn’t have an experience of being heard.

### 3.3. Controlling the child’s physical action with the picture schedule by allowing or restricting it

The picture schedule allowed children’s physical participation by giving the opportunity to point to the pictures and attach the “done” tags. The nurses generally moved from picture to picture quite fast (as in Extract 2), but in the following extract (Table 5), the nurse slows down, enabling the child to participate, even if in the beginning her quickness prevents the child from acting. The nurse (“Sofia”) and the child (“Niko”) sit and look at the schedule after they have completed the first activities of the day. Sofia has the schedule and one done tag in her hand. The tag box is between them.

**TABLE 5.** Data extract: Allowing the child to take physical action with the picture schedule.

1	Nurse	now that we have measured the height (.) there it was just measured (.) ((points at the measuring picture)) ((glances and points at the measuring place)) ((Niko glances at the measuring place only moving his eyes))
2		then you can put there like this that it’s @**done**@ (.) it’s already over (1.16) ((attaches the done tag on top of the picture, looks at Niko)) ((Niko turns to look at the tag box and moves his arm a little towards the box)) ((the nurse takes quickly one tag from the box))
3		then you can put it there since we’ve weighed (.) that’s already done (.) good ((passes the tag to Niko)) ((Niko attaches the tag quickly to the schedule)) ((Niko turns to the tag box) ((the nurse puts her hand in the box))
4	Nurse	and what else have we also done? ((takes the tag from the box and gives it to Niko))
5	Niko	((attaches the tag quickly to the schedule)) Nurse: well done
6	Niko	((takes quickly a new tag from the box right after attaching the previous one)) Nurse: yee:ah (.) good
7	Niko	((attaches quickly on top of the changing clothes picture)) Nurse: yee-ah (.) like that (.) ((points at the changing clothes picture))
8	Niko	((quickly takes a new tag from the box and attaches it to the schedule)) Nurse: and then the anaesthetic cream has also been applied (.) we have done ((points at the anaesthetic cream picture))
9	Nurse	this much already (.) good (.) like that (.) now we only have these left ((smiles and looks at Niko)) ((draws a circle in the air above the schedule)) ((Niko smiles mildly and looks at the schedule all the time))
10	Niko	((nods slightly looking still at the schedule))

This extract starts at the end of the hospital visit, when the nurse shows the picture schedule to Jani with the intention of attaching the last tags together. The situation builds up as a negotiation, where Jani four times expresses his intention to take the schedule home (lines 2, 11, 13 and 17). (Table 6) During his first suggestion, he also takes the schedule in his hand. In line 11, he grabs the schedule, but the nurse also holds on to it. In line 13, Jani repeats his intention while holding the schedule together with the nurse and looking at his mother.

**Table 6.** Data extract: Restricting the child to take physical action with the picture schedule.

1	Nurse	((turns the picture schedule for Jani to see))
2	Jani	I’ll take it with me ((takes the schedule in his hand))
3	Nurse	oh you’ll take it with you really= ((smiles mildly, takes the tag box in her hand))
4	Mother	ha-ha (.) we don’t take that either with [us
5	Nurse	it] probably stays here so that other children may also look at them (.) ((takes one tag from the box))
6		but do you wanna put these now on to it ((shows the tag to Jani and grabs the schedule))
7	Jani	yeaa (.) tere’s foud pitture ((releases from the schedule)) ((points at the food picture))
8	Nurse	yes there’s eating (.) you’ve eaten already (.) right? ((points at the food picture)) ((keeps the tag in front of the schedule)) ((attaches the tag))
9	Jani	Yeaa
10	Nurse	thaat way= ((moves her finger to the next picture))
11	Jani	I will take [it ((grabs the schedule, turns to mother’s direction))
12	Nurse	what] did we just do (.) look Jani (.) what did we just do? ((holds on to the schedule, tapping it with her other hand))
13	Jani	I will take it ((holds on to the schedule, looks at mother’s direction))
14	Mother	what did you just do? ((points at the schedule)) ((Jani turns to the schedule))
15		**(1 minute and 5 seconds of text excluded. During that time Jani has attached the rest of the tags guided by the nurse’s questions and the nurse has gone for a prize sticker for Jani)**
16	Mother	now all is done (.) let’s call it a day (.) let’s go home (1.13) finished ((draws a circle in the air above the schedule)) ((Jani turns his eyes to the schedule in his hands and scans it through))
17	Jani	I wanna have it ((turns his eyes to the side))
18	Mother	this we won’t take with us (.) this we leave [here (.) we have our own ((points at the schedule)) ((Jani makes an anxious sound))
19		pictures at home
20	Jani	huh] (- -) ((with a miserable tone))
21	Mother	we can’t take it ’cause it’s not ours (- -) ((Jani looks at the schedule))
22	Nurse	((comes back with the sticker box and passes it towards Jani)) there you go (.) you can choose from here (.) you can take home such as
23		this (.) this stays here (.) but you can take the sticker with you ((takes the schedule from Jani’s hands, puts it on the table))

Both the nurse and the mother resist Jani’s idea by supporting each other’s prohibitions. In addition, they give reasons for the refusal (the nurse in line 5 and the mother in lines 18–19 and 21). Furthermore, both the nurse and the mother try to direct Jani’s attention many times to other things such as attaching the done tags (lines 6, 12, 14) and selecting a sticker (lines 22–23). The nurse ends the situation by taking the schedule by force from Jani’s hand.

In summary, in this extract, adults do recognize the child’s expression as a relevant opening for a discussion and answer him by justifying their refusal. Yet they neither give him the opportunity to justify his wish, nor consider alternative ways of continuing the process with the schedule (e.g., taking a copy of the schedule or drawing the pictures). Instead, they use distraction techniques to avoid further discussing the subject. This time, the adults’ avoidance of negotiation with the child result in more than the child’s experience of not being heard: it concretely prevents the child from physically interacting with the preparation tool and being an active agent in his own preparation process.

## 4. Discussion

The study aimed at examining how the realization of the agency of children was enabled or hindered in the preparation process for an anaesthesia procedure with the help of a picture schedule. The results showed that although the characteristics of the picture schedule contributed to the process, the most crucial aspect was how it was used during interaction with the child (see also Capurso & Ragni, 2016). Communication aids do not automatically enhance the patient’s agency if they are used to promote the authority of the healthcare professionals (Chinn, 2020) or if they are not used for sharing the kind of information the patient needs (Mander, 2016), as studies on using Easy Read materials with adults with intellectual disabilities have demonstrated. In our study, sharing information reciprocally, responding to the child’s initiatives by negotiating and allowing the child to take physical action with the picture schedule enabled the realization of the agency of the children. This is possible when the adults listen to the children with all their senses, e.g., by observing their body language, and react to the children’s expressions as a valuable part of the interaction. In contrast, nonreciprocal information giving, responding to the child’s initiatives by sticking to the structure and restricting the child to take physical action with the picture schedule hindered it.

The results showed that the use of the picture schedule contributed positively to the realization of children’s agency. The pictures inspired the children, as also Thunberg et al. (2015) have noticed. This was demonstrated when the children used the picture schedule to take initiatives in communicating (getting information, expressing their views and making suggestions), in focusing on their own coping (structuring time, recalling the activities of the day) and in being active actors (participating physically by adding “done” tags and wanting to take the schedule home). In addition, when presenting the schedule, the nurses talked almost all the time directly to the children, instead of talking to the parents. Talking mainly with the parents is a common practice according to many studies (Hemsley et al., 2013; Hemsley et al., 2014; Oulton et al., 2015; Sharkey et al., 2016; Thunberg et al., 2016).

Despite the positive aspects, using the picture schedule was not trouble-free from the viewpoint of children’s agency. The nurses seemed to use the schedule mainly as a structuring tool, not as a reciprocal communication tool. This appeared in long sequences of giving information without offering the child the chance to participate, similarly to how Mitcheson and Cowley (2003) have described the use of a structured assessment tool. Even when nurses enabled the realization of the child’s agency in our data, it was about reacting to the children’s unprompted initiatives, not about nurses encouraging the children to ask questions or to express feelings. Neither did the schedule support that since it did not include pictures that would have directed the child to express his/her feelings or negotiate. Although getting information with the help of pictures is important for alleviating children’s anxiety (Vantaa Benjaminsson et al., 2015), it is not enough for children to be heard. Being heard might not mean that the child’s ideas will change the course of the day. But it makes possible for the adults to answer in a relevant way (from the child’s perspective) or at least let the child know they are trying to understand him. And even if enabling the realization of the child’s agency does not mean fulfilling all of his wishes, it should probably mean fulfilling those wishes that indicate the child’s willingness to participate actively in his preparation process. Pointing at the pictures also allowed the nurses to avoid using the names of the unpleasant activities, instead only referring to the pictures with pronouns.

We studied only the micro-level interaction of the nurses and children, but it is important to notice that several macro-level factors might also contribute in the interaction practices of the nurses. The organizational culture affects the manner of interaction of individual nurses, for example, if the nurses are socialized in the “practitioner as expert” thinking (Mitcheson and Cowley, 2003). The organizational structures may also force nurses into routines that direct patients into a position where they have very few opportunities to influence the way discussion progresses (Jones, 2003). In addition, behind the organizational cultures are paradigms that guide professionals’ thinking (Handberg & Voss, 2018). One part of the nursing paradigm, in this case, is the image of a child (Karlsson, 2020). In the context of a children’s neurological ward, nursing practices have been mostly found to position children as vulnerable individuals who need protection and are more like objects for the professionals’ procedures than active subjects (Olli et al., 2014; Olli et al., 2021), as did the preparation practices in this study. The image of a professional is also worth considering (Olli et al. 2012). The practices were mainly based on giving information or structuring the activities, not on a reciprocal process based on a perspective the child expressed. The nurses did not actively ask for the child’s perspective, but sometimes they did respond to the children’s persistently expressed initiatives in a way that enabled the realization of the child’s agency.

### 4.1. Relevance to clinical practice

According to our findings, it is important that the features of the preparation tool encourage reciprocal interaction in informing and in responding to the children’s initiatives. The flexibility of the tool, such as the opportunity for the child to add or remove pictures or change their order in a schedule, would contribute positively to the child’s agency. Another important addition would be pictures that allow the expression of feelings, as was done in the study by Vantaa Benjaminsson et al. (2015), as well as pictures that encourage the child’s questions.

The tool should also include opportunities for the child to participate physically. Our study demonstrated how children might consider certain matters significant that adults seem to perceive as small details. For instance, for an adult, it might seem irrelevant who puts the tag on the schedule, but for the children it seemed to be important to get the chance to do it.

In addition, the practices of using the tool should be considered. If the picture material were sent to their home beforehand (see Capurso & Ragni, 2016; Vantaa Benjaminsson et al., 2015), the information could be given in a more child-originated way, such as by letting the child ask about the pictures or asking the child’s what he/she understands about them. Sending the picture material to the child’s home would enable using it after the hospital visit as well, as the children in our study wanted to do.

The preparation practices should also include enough time for the child’s initiatives and physical participation. Our study illustrated that inserting pauses in speech and actions and giving only one minute more might be enough for a child. Having more time for communication when the child has complex communication needs has been shown to be important in previous studies as well (Finke et al., 2008; Hemsley & Balandin, 2014). Since analysing the video material proved to be useful in this study, video clips from different preparation situations could be used when training nurses to develop their practices. Our recommendations aim at preparation as a dialogical process based on respecting the children’s perspectives and positioning them as agents (see Lindberg and von Post, 2005, 2006). This would require reflection on the image of a child and the image of a professional, both in the education of nurses and in work communities. We also suggest using the expression “helping the children prepare” instead of the paternalistic expression “preparing children”.

In the future, it would be useful to study the wider consequences of the process of helping the children prepare and of the ways it enables or does not enable the realization of the child’s agency. If anaesthesia procedures may cause problems in the child’s life after one year (Kain et al., 1996), what kinds of positive long-term consequences might a process that enables the child’s agency have? The long-term consequences will be different if the aim of the preparation process is for children to arrive happily for the procedure and be able to feel proud of themselves during the procedure and afterwards (Lindberg and von Post, 2005, 2006), in contrast to only aiming to lower their anxiety. In addition, it would also be important to examine the role of parents in enabling or hindering the child’s agency, since it has been shown that sometimes parents speak on behalf of the child even when a nurse tries to speak directly to the child (Hemsley et al., 2014). Finally, we suggest that more studies are conducted with disabled children, since “what is good for people with disability is good for everyone”, as Thunberg et al. (2015) have stated.

### 4.2. Strengths and limitations

The transferability of our findings was strengthened by the rich data that included interaction sequences from every child and every nurse of both situations that enabled the realization of the child’s agency and those that hindered it. Therefore, the realization of the child’s agency cannot be explained by the child’s or the nurse’s characteristics, but by the different interaction practices. Although the participants of the study were disabled children, there is no reason to assume that the results would not apply to other children, since the findings reveal some common patterns in interaction. The results can be used in assessing preparation methods with all kinds of children and even adults – especially with elderly people – since the findings are not related to the child’s age or impairment, or to the content of the procedure.

Applied conversation analysis offered a useful method for analysing multimodal communication. Analysing nonverbal modalities appeared to be crucial from the perspective of children’s agency. In CA, the interpretation is based solely on what can be seen in the data, where every turn is interpreted in the context of the previous turn (Heritage, 2001). Nevertheless, the preunderstanding of the analysers always influences what they are able to see in the data. In our study, the first author met the children before the data collection during the assent process. Asking for their assent and seeing them in their home environment enabled her to approach them as active agents also when analysing the data (Olli, 2019). The different disciplines of the authors (nursing science, special education, childhood studies and disability studies) also contributed versatile perspectives to the article.

Video recording has often been seen as a limitation because it might affect the participants’ behaviour. It is possible that the participants were nervous because of the video recording and therefore not able to give their best. Alternatively, they might have tried more actively to give their best. However, the aim of our study was not to prove what nursing interaction is like, but to demonstrate what consequences different kinds of interaction practices might have for the child’s agency. One of the biggest challenges in our study was transcribing the video data, especially concerning nonverbal communication and not every nuance might have been noted in the transcription. Consequently, we used the transcriptions and also repeatedly watched the recordings during the analysis and writing of this article.

## 5. Conclusion

This study shows how disabled children’s right to be heard is not always implemented optimally in healthcare and the professional-originated idea of “preparing the child” is more dominant in the practices than the idea of “helping the child prepare”, which emphasizes the child’s perspective. This appeared in our data both in the situations when the realization of the child’s agency was enabled and when it was not, since the nurses did not actively offer children opportunities to express their views or ask questions. They did answer, though, when the children asked them, or other ways took their space themselves. The study provides material to use in reflecting on whether one’s tools and practices for helping the children prepare for a procedure give children the opportunity to influence information sharing, to have their initiatives taken seriously or to participate bodily. This interactional perspective is widely neglected in studies concerning children’s preparation processes. In addition, in order to develop the nursing interaction, it would be essential to discuss the image of a child held by the nurses and its consequences for nursing practices and the organizational cultures of hospitals.

## Appendix 1. Transcription symbols

### Pauses

(.) Brief, untimed pause, less than 1 second.

(1.0) The length of pauses of 1 second or more.

### Overlap

[Start of simultaneous talk.

] End of simultaneous talk.

= No discernible pause between the end of a speaker’s utterance and the start of the next utterance.

text- Interruption (unfinished word).

### Rhythm

: An extension of the preceding vowel sound.

### Strength of voice

TEXT Text that is spoken more loudly than the surrounding talk.

text Text is spoken with emphasis.

### Other symbols

ha-ha Laughing.

@text@ Changing one’s typical voice.

### Comments from the transcriber

(- -) Incomprehensible.

((text)) Comments from the transcriber.
